# Supplementary material for: The prevalence and factors associated with common mental health conditions among female sex workers in Dar es salaam, Tanzania
Source: PLOS Ment Health. 2026 Apr 15;3(4):e0000523. doi: 10.1371/journal.pmen.0000523 (PMC13082590; doi:10.1371/journal.pmen.0000523)
Supplement: Attachment — Submitted filename: Response to Reviewers.docx [file pmen.0000523.s005.docx]

**Edwin Ngula Luguku,**
Dar es salaam,

Tanzania.
March 6, 2026.

**Editor-in-Chief**
PLOS Mental Health

**Dear Editor,**

I am pleased to re-submit our manuscript entitled ***“The prevalence and factors associated with common mental health disorders among female sex workers in Dar es Salaam, Tanzania”*** for consideration as a research article in *PLOS Mental Health*.

We would like to extent our gratitude for the review process by the editorial team and the two reviewers, particularly finding merit in our paper and the valued comments that aim at improving the quality of our submission. Hereunder is a tabular description of our response to the reviewers’ comments.

| **S/N** | **Comments from the reviewer** | **Response** | **where changes have been made** |
| --- | --- | --- | --- |
| 1. | **Data availability statement.** | We have revised the Data Availability Statement to clarify that the dataset cannot be publicly shared because it contains sensitive human participant information. We also clarified that data access is regulated by the Tanzania National Health Research Ethics Committee (NatHREC), which does not permit public sharing or transfer without prior approval. In line with your recommendation, we have added the institutional contact details for data requests in the manuscript. | - |
| 2. | **PLOS’ questionnaire on inclusivity in global research** | We have completed and uploaded the PLOS’ questionnaire on inclusivity in global research  A subsection referencing to the supplemental material (S2 Checklist) has been added. | Uploaded document  122 - 124 |
| 3. | **Recruitment of Respondents**  It would be appreciated if the authors could elaborate in detail on the recruitment process. Specifically, the manuscript should clearly describe how respondents were identified, approached, and enrolled in the study, as well as indicate who was responsible for conducting the recruitment. | We have included a clearer description of the recruitment processes, addressing how respondents were identified, approached and enrolled. We have clearly identified that one of the researchers with support from an NGO supporting community entry were engaged in the recruitment processes.  We have also uploaded a figure as a supplemental material to provide further context. (S1 Figure) | 101 - 113 |
| 4. | **Data Collection**  The authors are requested to provide a detailed account of the data collection procedures followed in this study. This should include the step-by-step process undertaken during data collection. The manuscript states that, to “ensure privacy, interviews took place in a designated room around the hotspot.” It is unclear whether the term interviews refers to the administration or completion of questionnaires. Alternatively, it raises the possibility that a mixed-methods research design was employed. The use of the term interviews is therefore confusing, particularly because the study is presented as quantitative in nature. Clarification on this issue is required. | We thank the reviewer for this observation. We have revised the manuscript to clarify that data were collected through a researcher-administered structured questionnaire in a private room near the hotspot. We have also replaced the term “interview” with “questionnaire administration” to avoid confusion and to clearly reflect the quantitative design of the study.  We have also uploaded a figure as a supplemental material to provide further context. (S1 Figure) | 114 - 121 |
| 5. | **Study Setting**  It is recommended that the study setting be described more comprehensively. This should include relevant contextual and environmental details, together with a clear justification for why this particular setting was selected for the study. | We have revised the Study Setting section to provide a more detailed description of the study context, including the urban hotspot-based environment in which female sex workers operate in Dar es Salaam, the three municipalities included in the study, and the rationale for selecting this setting. | 128 - 134 |
| 6. | **Recommendations**  The study’s recommendations are primarily directed at researchers, advocates, and policymakers. While these stakeholders are important, such recommendations may require a longer period to implement due to their higher-level focus. It is therefore suggested that the study also propose practical and context-specific recommendations that can be implemented immediately at a grassroots level by sex workers themselves, as well as by their managers and relevant associations. Immediate, actionable interventions are especially necessary to address the reported suicidal ideation and depressive symptoms. | We thank the reviewer for this important suggestion. We have revised the recommendations section to include immediate, practical, and context-specific actions that can be implemented at grassroots level by female sex workers, hotspot managers, and community-based organizations, particularly to support early identification, peer support, and referral for women experiencing depressive symptoms or suicidal ideation or recent violence. | 450 - 456 |
| 7. | **Reference list.** | Reviewed references for completeness.  Added one new reference under the study setting (25)  Updated one reference (31) |  |

Thank you for considering our manuscript for publication. We look forward to your feedback.

Sincerely,

**Edwin Ngula Luguku**
